# Transcriptomic analysis of *Verbena bonariensis* roots in response to cadmium stress

**DOI:** 10.1186/s12864-019-6152-9

**Published:** 2019-11-20

**Authors:** Meng-qi Wang, Zhen-yu Bai, Ya-fang Xiao, Yan Li, Qing-lin Liu, Lei Zhang, Yuan-zhi Pan, Bei-bei Jiang, Fan Zhang

**Affiliations:** 1 Department of Ornamental Horticulture, Sichuan Agricultural University, Chengdu, Sichuan 611130 People’s Republic of China; 2 Institute of Agro-Bioengineering and College of Life Sciences, Guizhou University, Guiyang, Guizhou 550025 People’s Republic of China

**Keywords:** *Verbena bonariensis*, Cadmium stress, RNA-Seq, Physiological changes, Molecular mechanism

## Abstract

**Background:**

Cadmium (Cd) is a serious heavy metal (HM) soil pollutant. To alleviate or even eliminate HM pollution in soil, environmental-friendly methods are applied. One is that special plants are cultivated to absorb the HM in the contaminated soil. As an excellent economical plant with ornamental value and sound adaptability, *V. bonariensis* could be adapted to this very situation. In our study, the Cd tolerance in *V. bonariensis* was analyzed as well as an overall analysis of transcriptome.

**Results:**

In this study, the tolerance of *V. bonariensis* to Cd stress was investigated in four aspects: germination, development, physiological changes, and molecular alterations. The results showed that as a non-hyperaccumulator, *V. bonariensis* did possess the Cd tolerance and the capability to concentration Cd. Under Cd stress, all 237, 866 transcripts and 191, 370 unigenes were constructed in the transcriptome data of *V. bonariensis* roots. The enrichment analysis of gene Ontology (GO) and Kyoto Encyclopedia of Genes and Genomes (KEGG) pathway revealed that differentially expressed genes (DEGs) under Cd stress were predominately related to cell structure, reactive oxygen species (ROS) scavenging system, chelating reaction and secondary metabolites, transpiration and photosynthesis. DEGs encoding lignin synthesis, chalcone synthase (CHS) and anthocyanidin synthase (ANS) were prominent in *V. bonariensis* under Cd stress. The expression patterns of 10 DEGs, validated by quantitative real-time polymerase chain reaction (qRT-PCR), were in highly accordance with the RNA-Sequence (RNA-Seq) results. The novel strategies brought by our study was not only benefit for further studies on the tolerance of Cd and functional genomics in *V. bonariensis*, but also for the improvement molecular breeding and phytoremediation.

## Background

HM pollution in soil has long jeopardized the sustenance of plants. As a kind of poisonous HM, Cd served as a botanic destroyer [1]. Absorbed by roots, HMs in soil are transported to the aboveground parts of plants. The accumulation of HMs hampers the growth and development of plants. Accordingly, through food chain, this toxic matter endangers animals and human.

The excessive concentration of HMs has a severe impact on the growth, plasma membrane permeability, physiological and biochemical processes and nutritional status of plants [2]. The increased production of ROS under HM stress damages cell membranes, decomposes nucleic acids and declines photosynthesis of plants [3, 4]. ROS ruins balance between production and the activity of antioxidative system. Cd disrupts the growth and development of the plant by trespassing. The chelation is in response to HM stress in the plants. There are four main chelating agents in plants, including phytochelatin (PC), metallothionein (MT), organic acid and amino acid [5]. PCs plays an important role in detoxification of intolerable HMs to balance the internal metal elements. It is synthesized non-translationally from reduced glutathione (GSH) in a transpeptidation reaction catalyzed by the enzyme PC synthase. The sensitivity of secondary metabolites to HM is species-specific [6]. The diversity as well as functions of soil microbial community structure were determined by the generation of root exudation in plants [7]. The study of ‘Plants call for support’ posed a hypothesis that the alteration of pollution-induced root exudation aided the botanical selection of microbial communities to reduce the stress of the pollution to the root system [8]. It is suggested that to adapt to HM stress, metabolism is modified, along with the production of secondary metabolites, in plant tissues [9].

The RNA-Seq platform was used for the detection of plants under Cd stress. Gu et al. [10] investigate the transcriptome in *Iris lactea var. chinensis* under Cd and Pb stresses. Yongkun et al. [11] conducted a transcriptome analysis of Cd responses in *Phytolacca americana L.* Gao et al. [12] demonstrated that several genes involved in modifying cell wall and translocating metal ion had higher expressed levels in *S. alfredii Hance* shoots than that in non-hyperaccumulating ecotype shoots under exposing Cd stress. Similar results were also reported in *Populus × canescens* [13], *Noccaea caerulescens* [14], *N. caerulescens* [15], *Viola yedoensis Makino* [16] and *Arabidopsis thaliana* [17] using transcriptome analysis.

Due to strong adaptability, vigorous growth and highly ornamental value of *V. bonariensis*, especially with the popularity of sightseeing farms, it owned the potential in large scale cultivation. Therefore, the rehabilitation ability of *V. bonariensis* under HM stress secured the spotlight. In this study, we investigated the germination, morphologic and physiologic response along with the Cd^2+^ accumulation in *V. bonariensis*. In addition, a high-throughput sequencing technique was applied to construct the transcriptome database of *V. bonariensis* under Cd stress. The molecular mechanism of transportation and detoxification of Cd was analyzed based on sequence annotation. This study would made contribution to the discovery of potential Cd defensive strategies in *V. bonariensis*.

## Results

### *The germination and cd accumulation in V. bonariensis under different cd concentration stress*

Table 1 showed that the influence of Cd^2+^ on the seed germination depended on its concentration. Germination rate and Germination index (GI) was higher at 20 mg/L than that of controlling groups. In 20 mg/L and below, vigor index (VI) and fresh weight were promoted on various degrees. At 14 d, all the seedlings treated with over 50 mg/L concentrations of Cd died.

**Table 1:** Effect of Cd concentration on germination of *Verbena bonariensis*

7 d					14 d
Concentration (mg/L)	Germination rate (%)	Germination index	Vigor index	fresh weight per plant (mg)	Survival rate (%)
0	97.78 ± 1.92^ab^	25.33 ± 0.29^abc^	0.0532 ± 0.0020^abc^	2.10 ± 0.10^abc^	97.78 ± 1.92^ab^
5	97.78 ± 1.92^ab^	25.67 ± 0.29^ab^	0.0573 ± 0.0056^a^	2.23 ± 0.23^a^	97.78 ± 1.92^ab^
10	97.78 ± 1.92^ab^	25.67 ± 0.58^ab^	0.0574 ± 0.0050^a^	2.23 ± 0.15^a^	97.78 ± 1.92^ab^
20	100.00 ± 0.00^a^	26.50 ± 0.50^a^	0.0565 ± 0.0025^ab^	2.13 ± 0.06^ab^	98.89 ± 1.92^a^
50	97.78 ± 1.92^ab^	25.50 ± 1.50^ab^	0.0494 ± 0.0043^bcd^	1.93 ± 0.06^bcd^	46.67 ± 6.67^b^
100	96.67 ± 5.77^ab^	24.83 ± 0.77^bcd^	0.0471 ± 0.0011^cd^	1.90 ± 0.10^bcd^	0.00 ± 0.00^c^
150	95.56 ± 1.93^ab^	24.00 ± 1.00^cd^	0.0449 ± 0.0069^d^	1.87 ± 0.21^cd^	0.00 ± 0.00^c^
200	92.22 ± 5.09^b^	23.50 ± 0.50^d^	0.0423 ± 0.0009^d^	1.80 ± 0.00^d^	0.00 ± 0.00^c^

Note: Data represent means±SE of three replicates. The different letters above the columns express significant differences (*P* < 0.05) on the basis of Duncan’s multiple range test

**Fig. 1:**
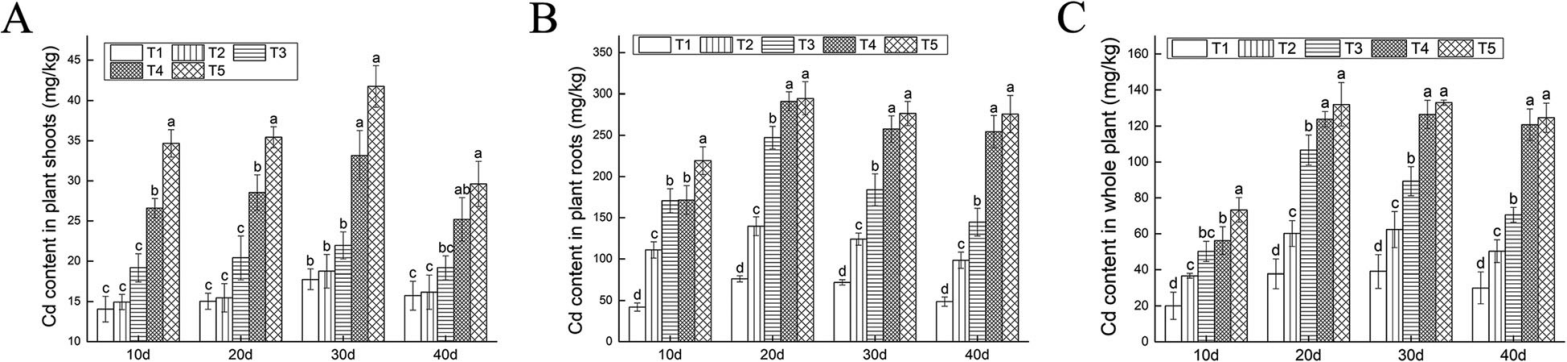
Enrichment of Cd in plants. **a** Cd content in plant shoots. **b** Cd content in plant roots. **c** Total Cd content in *Verbena bonariensis*. A total of 50 mg/kg (T1), 100 mg/kg (T2), 200 mg/kg (T3), 300 mg/kg (T4), and 400 mg/kg (T5) were set up for 5 Cd concentrations. Standard error of the mean for three repetitions is represented by the error bars. The different letters above the bars indicate the significant difference at P < 0.05 among the different treatments. The same below

**Fig. 2:**
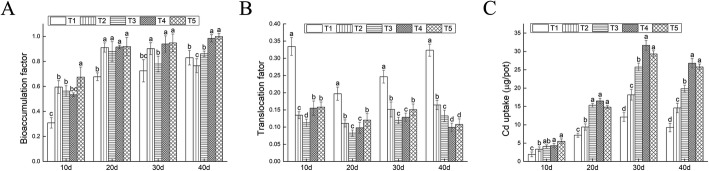
The impacts of Cd in soil on the bioaccumulation factor, translocation factor and Cd uptake of *Verbena bonariensis*. **a** bioaccumulation factor of Cd in roots. **b** translocation factor of Cd in *Verbena bonariensis*. **c** Cd uptake by *Verbena bonariensis*

The contents of Cd in the shoots and roots increased with Cd concentration and time, while the Cd contents in roots were significantly higher than those in the shoots (Fig. 1a, b). When the Cd concentration in the soil increased to 400 mg/kg (T5) for 30 d, Cd content reached the maximum, 133.11 mg/kg, in whole plants (Fig. 1c). According to Fig. 2a, the minimum bioaccumulation factor (BCF) (at the root of the plant) was in proportion to duration and concentration of Cd stress. The range of variation is 0.309 to 0.999. According to Fig. 2b, translocation factor (BTF) reached to the maximum (0.3344) at the 50 mg/kg Cd concentration. The absorption of HMs is one of the signaling indicators for the HM purifications of the hyperaccumulator. It could be found in Fig. 2c that under all concentration Cd absorption reached its peak at 30 d. The maximum is 31.66 μg/pot in the 300 mg/kg (T4).

### *The morphological and physiological changes of V. bonariensis under 100 mg/kg cd stress*

According to the measurement of various morphological (Additional file 1: Figure S1; Additional file 2: Figure S2) and physiological (Additional file 3: Figure S3) indexes in the prophase, the seedlings treated in 100 mg/kg solution was selected for RNA-Seq. The morphological and physiological changes of the plants treated respectively under the control group (CK) and 100 mg/kg Cd concentration for 20 d were compared.

**Fig. 3:**
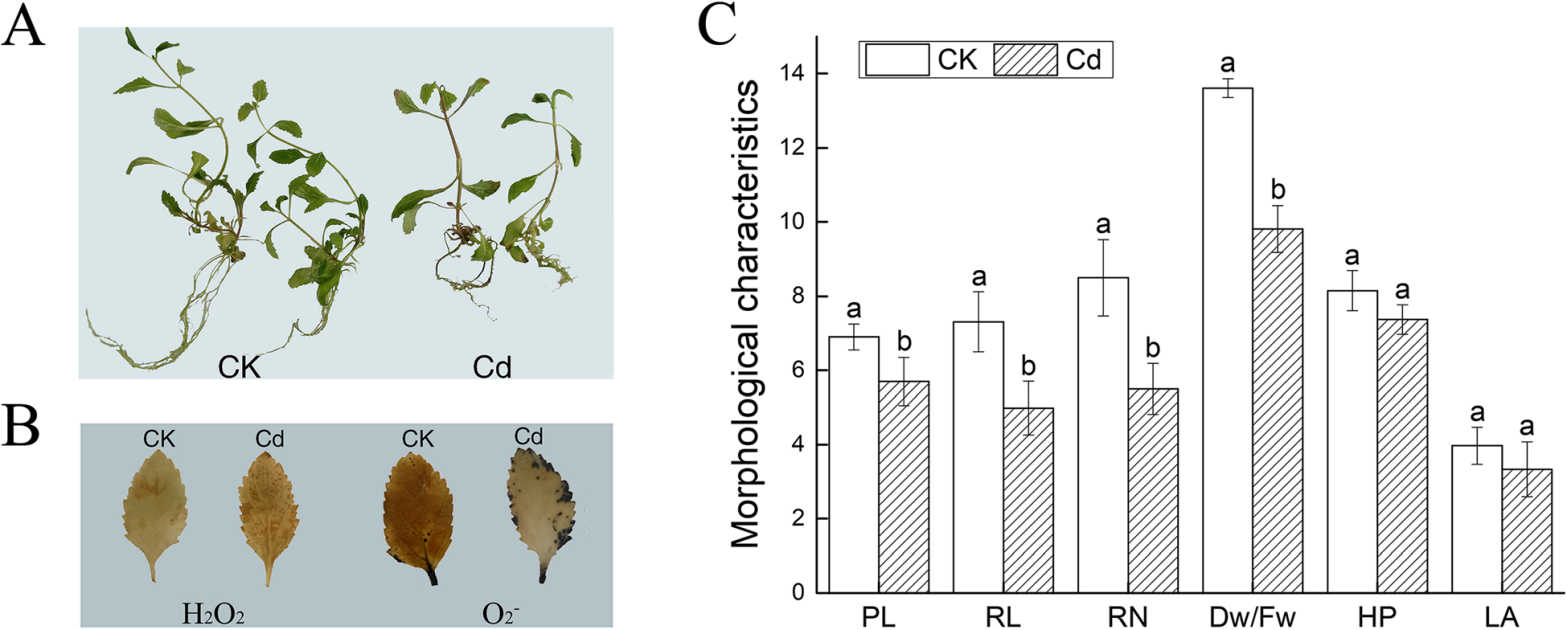
Effects of Cd stress on external morphology and active oxygen metabolism in *Verbena bonariensis*. **a** The comparison of vitro morphology of plants from CK and Cd treated. **b** The comparison of ROS staining of leaves from control and Cd-treated *Verbena bonariensis* plants. **c** The indexes of morphological characteristics. Plants were grown with 100 mg/kg Cd for 20 d

**Fig. 4:**
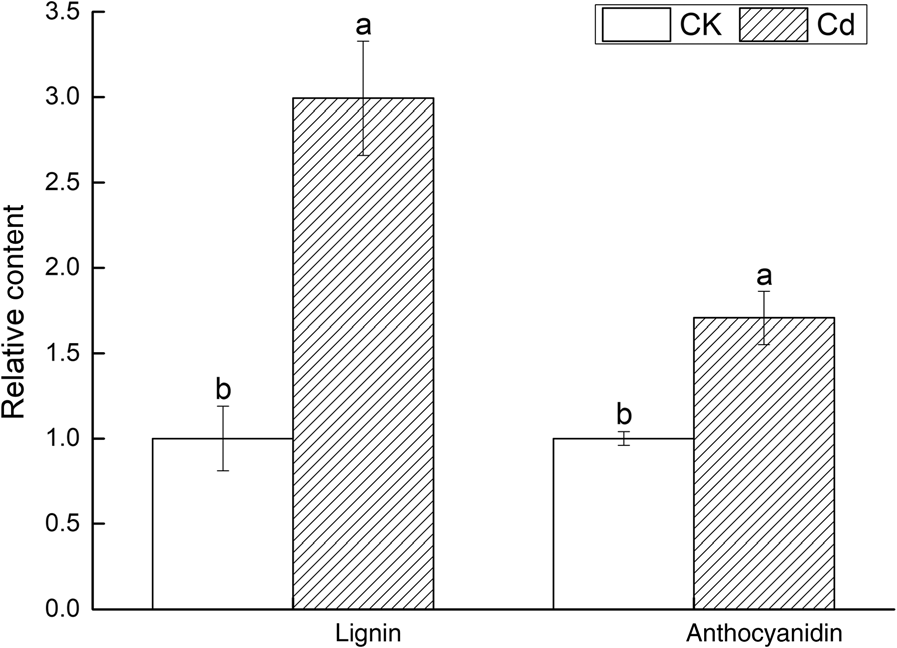
The relative content of lignin and anthocyanidin

**Fig. 5:**
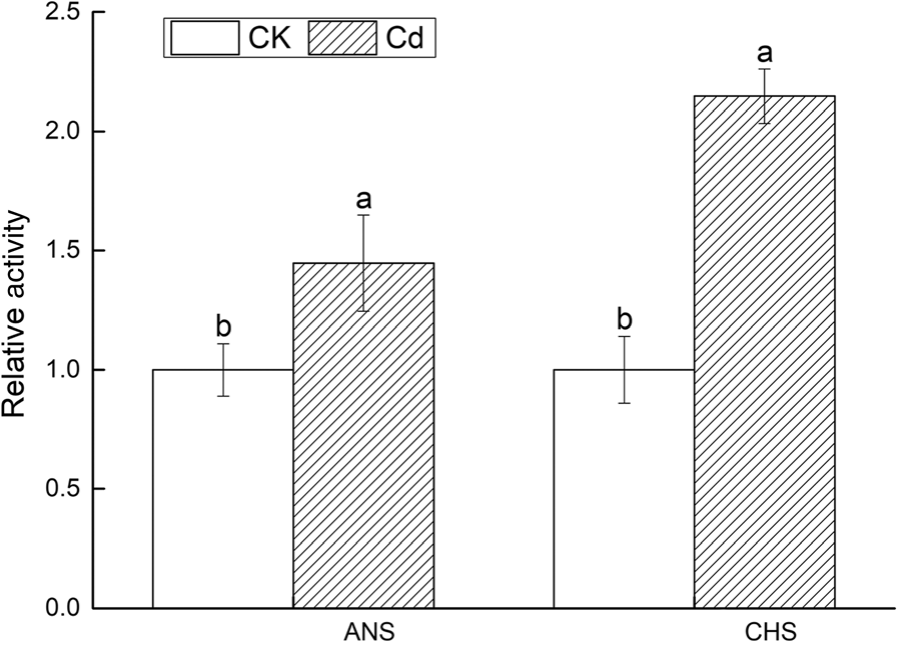
The relative activity of ANS and CHS

**Fig. 6:**
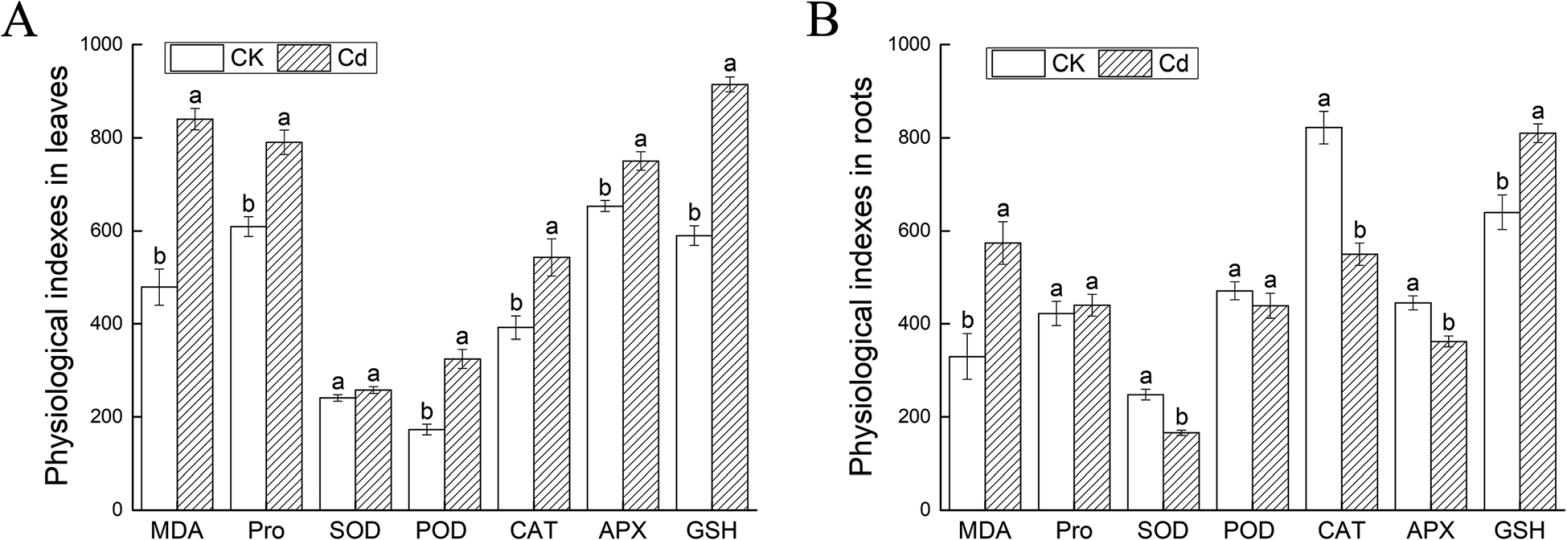
Effects of Cd on physiological indexes of *Verbena bonariensis*. **a** The changes of leaves under Cd stress; **b** The changes of roots under Cd stress. SOD and APX activity as u·g^−1^, POD activity was expressed as u·g^− 1^·min^− 1^, CAT as 10^− 1^·u·g^− 1^·min^− 1^, GSH as 10^− 2^·u·g^− 1^ FW, proline as ng·ml^− 1^ and MDA as 10^− 1^·nmol·L^− 1^

**Fig. 7:**
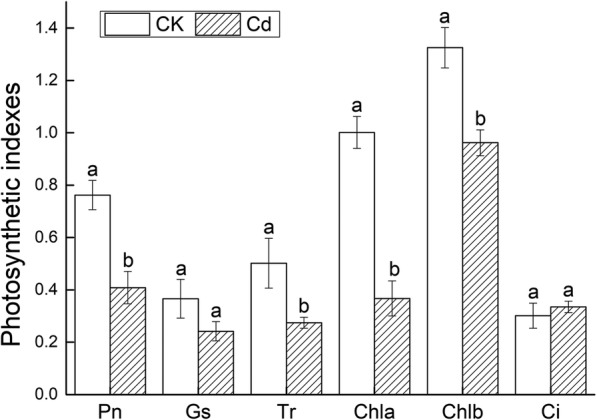
Effects of photosynthesis under Cd stress in *Verbena bonariensis* leaves. Pn and Tr were expressed as umol·m^− 2^·s^− 1^, Gs was expressed as 10^− 1^·mol·m^− 2^·s^− 1^, Ci as ml·L^− 1^, Chla and Chlb as mg·g^− 1^

The dwarf plants, yellow leaves, slight dark roots were inspected on Fig. 3a. A large amount of H_2_O_2_ and O_2_^−^ produced in leaves were observed on Fig. 3b. The petiole length (PL), the root length (RL), number (RN) and dry to fresh ratio (Dw/Fw) were significantly reduced by 17.39, 31.87, 35.29 and 27.92%, respectively. The height of upper part (HP) and leaf area (LA) declined slightly. All morphological indexes declined (Fig. 3c). The content of lignin and anthocyanidin (Fig. 4), the activity of ANS and CHS were higher than that of the control (Fig. 5). Cd^2+^ increased the content of malondialdehyde (MDA) and proline (PRO) as well as the GSH activity in leaves and roots. The superoxide dismutase (SOD), peroxidase (POD), catalase (CAT) and ascorbate peroxidase (APX) were elevated in leaves while decreased in roots under Cd stress (Fig. 6). Net photosynthetic rate (Pn), stomatal conductance (Gs), transpiration rate (Tr), Chlorophyll a (Chla) and chlorophyll b (Chlb) decreased on various degrees. CO_2_ concentration (Ci) slightly increased (Fig. 7).

### Sequence analysis and assembly

Large amounts of data were produced by sequencing the two libraries (CK and Cd) of *V. bonariensis* with the Illumina HiSeq 2500. After data filtering, a total of 55, 962, 351 and 61, 462, 567 clean reads with 93.33 and 93.36% Q30 bases were selected for the CK and Cd libraries, respectively. With the Trinity program, all 237, 866 transcripts and 191, 370 unigenes with an average length of 1103 bp and 1298 bp were constructed in total. Data files obtained by Illumina HiSeqTM was submitted to the NCBI database with accession number GSE113569.

### Sequence annotation and classification

Compared with the public seven databases, a total of 153, 895 (80.41%) annotative unigenes were obtained. The successful rate of the functional annotation in the seven databases was shown in (Additional file 4: Figure S4). *Sesamum indicum* (97,567 unigenes) offered a prior similarity with *V.bonariensis*, then did the *Erythranthe guttata* (20,692).

Using the GO annotation database, a total of 101,415(52.99%) unigenes were annotated and there were 50.98% in Biological process (BP), 35.44% in Cellular component (CC), and 13.57% in Molecular function (MF) (Additional file 5: Figure S5). In all three data sets, ‘cellular process’, ‘metabolic process’ and ‘single-organism process’ were the most highly represented under BP; ‘cell’, ‘cell part’ and ‘organelle’ terms were dominant in CC, and ‘binding’ and ‘catalytic activity’ were the most significant terms in the MF. Using the KEGG database, a total of 57,061 unigenes were grouped into five branches. Among these pathways, ‘Carbohydrate metabolism’ was the group with the greatest number of genes (5164, 9.06%), followed by ‘Translation’ (4284, 7.50%) and ‘Folding, sorting and degradation’ (3767, 6.60%).

### Analysis of GO term and KEGG pathway involving DEGs

In order to further understand the alteration in gene expression of *V. bonariensis* responding to Cd stress, differential expression analysis with DEGseq was performed. All 23, 424 DEGs were obtained, of which 12,558 were up-regulated while 10,866 were down-regulated under Cd treatment.

A total of 16,580 DEGs in *V. bonariensis* were enriched in 60 GO terms. BP, CC and MF accounted for 55.28, 12.83 and 28.65%, respectively. Among the top 15 significantly enriched GO terms for DEGs, seven GO terms were related to cell wall (Table 2).

**Table 2:** The top-15 significant enriched GO terms involving DEGs under Cd stress

Description	Term_type	Up-regulated DEGs number	Down-regulated DEGs number
structural constituent of cell wall	molecular_function	101	3
oxidation-reduction process	biological_process	1354	802
oxidoreductase activity	molecular_function	1337	773
plant-type cell wall organization	biological_process	111	5
plant-type cell wall organization or biogenesis	biological_process	111	5
catalytic activity	molecular_function	5244	3879
cell wall	cellular_component	160	35
external encapsulating structure	cellular_component	181	48
cell wall organization	biological_process	145	21
heme binding	molecular_function	333	160
tetrapyrrole binding	molecular_function	333	167
external encapsulating structure organization	biological_process	148	21
cell wall organization or biogenesis	biological_process	187	40
cell wall biogenesis	biological_process	141	20
single-organism metabolic process	biological_process	2820	1954

A total of 8600 DEGs were assigned to 124 KEGG pathways. Table 3 showed the top-ten significant up-regulation and down-regulation pathways involving DEGs, respectively. In top-ten up-regulated pathways, the ‘glutathione metabolism’ was the most significantly up-regulated pathway. All 133 DEGs were up-regulated and accounted for 76% of all DEGs of this pathway. There were three pathways relating to organic acid metabolism in top-ten up-regulated pathway, including ‘Citrate cycle (TCA cycle)’ (88 up- and 10 down-regulated DEGs), ‘Glyoxylate and dicarboxylate metabolism’ (82 and 40) and ‘alpha-Linolenic acid metabolism’ (60 and 23). The ‘Photosynthesis-antenna proteins’ and ‘photosynthesis’ were the first two significantly down-regulated pathways. In ‘Photosynthesis-antenna proteins’ pathway, all 76 DEGs (75 down- and 1 up-regulated DEGs) were related to the light-harvesting chlorophyll protein complex (LHC). Eighteen DEGs were related to Lhca, while 58 DEGs were involved in Lhcb. In ‘Photosynthesis’, only 9 genes in all 78 DEGs were up-regulated. In addition, the secondary metabolism pathway was worth mentioning. In ‘phenylpropanoid biosynthesis’, all the 18 DEGs associated with lignin synthesis was up-regulated (Additional file 6: Table S1). CHS (5 DEGs) and ANS (9) were related to flavonoid biosynthesis (Additional file 7: Table S2).

**Table 3:** The top-ten significant enriched KEGG pathways involving DEGs under Cd stress

Regulation	Pathway term	Rich factor	FDR	Gene number
Up-regulated	Glutathione metabolism	0.223529	2.42E-10	133
	Citrate cycle (TCA cycle)	0.226221	3.80E-07	88
	Phenylpropanoid biosynthesis	0.179342	9.83E-07	158
	Proteasome	0.233974	1.13E-06	73
	Carbon fixation in photosynthetic organisms	0.180113	0.000261	96
	Glycolysis / Gluconeogenesis	0.157366	0.001086	141
	Flavone and flavonol biosynthesis	0.377778	0.00125	17
	Galactose metabolism	0.169091	0.001704	93
	Glyoxylate and dicarboxylate metabolism	0.172632	0.002082	82
	alpha-Linolenic acid metabolism	0.180723	0.004917	60
Down-regulated	Photosynthesis - antenna proteins	0.675676	2.18E-35	75
	Photosynthesis	0.345	1.82E-19	69
	Glycerophospholipid metabolism	0.16109	6.45E-12	130
	Glycerolipid metabolism	0.179704	6.71E-10	85
	Carotenoid biosynthesis	0.193133	3.94E-06	45
	Ether lipid metabolism	0.181818	3.94E-06	50
	Circadian rhythm - plant	0.178439	9.42E-06	48
	Starch and sucrose metabolism	0.112982	4.09E-05	161
	Vitamin B6 metabolism	0.301587	4.65E-05	19
	Plant hormone signal transduction	0.108998	0.000395	149

### qRT-PCR

**Fig. 8:**
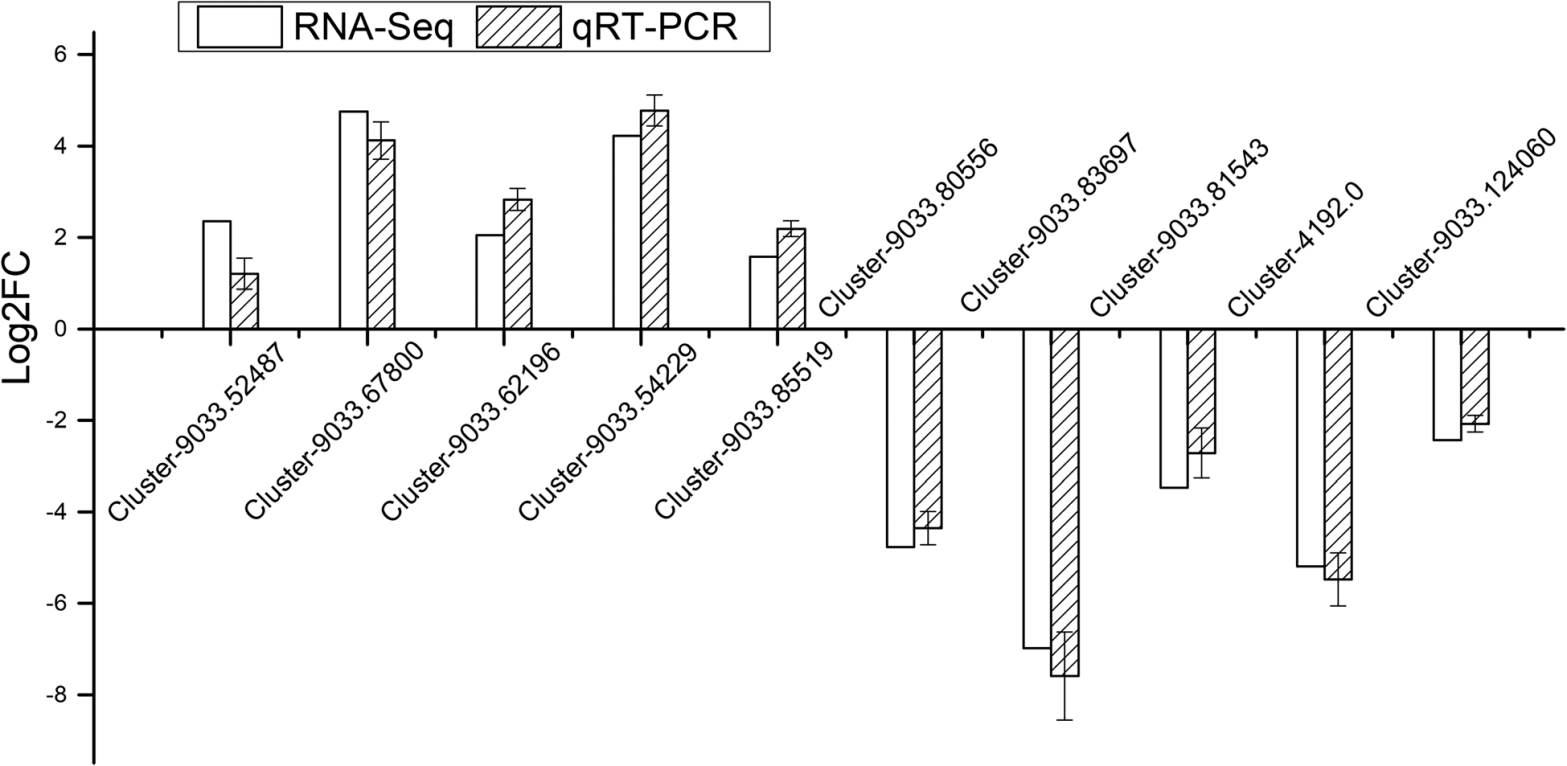
Validation of RNA-Seq results using qRT-PCR. The gene primers used for RT-qPCR analysis are shown in Additional file 7: Table S2. Standard error of the mean for three repetitions is represented by the error bars

To confirm the reliability of high-throughput sequencing results, ten DEGs were selected and analyzed for qRT-PCR. It proved that the fold variation between RNA-Seq expression and qRT-PCR analyses was almost the same (Fig. 8).

## Discussion

### The germination and morphological alteration of *V. bonariensis* under differential cd stress

During germinal and individual development, seeds was sensitive to environmental stress [18]. Therefore, the study on this stage reflected the tolerance to these stress in plants. Previous studies have demonstrated that 10 mg/L Cd concentration severely affected the germination of *Medicago sativa* [19]. *Coreopsis drummondii* and *Impatiens walleriana Hook. f.* seeds, compared with the controlling group, the germination rate of experimental group was reduced by about 50% [20]. Our results showed that the threshold Cd concentration on *V. bonariensis* germination was about 50 mg/L. Cd solutions within 20 mg/L concentration promoted the germination and growth on seedlings.

The growth and morphology alteration served as the basic adaptation mechanisms. The roots were suffered primarily from HMs in soil sites. Botanical growth was hindered, pigmentation, lateral root numbers, root activity were lessened. The absorption of water and nutrient utilization were disturbed [21]. With the HM ions shifted to shoot, the symptoms of toxicity altered: plant dwarfism, leaf chlorosis, reduced biomass, inhibited photosynthesis occurred, eventually death happened [22]. Under Cd stress, these changes were present in *V. bonariensis* (Fig. 3). Under Cd stress the roots elongation was severer inhibited than in the aboveground part of *V. bonariensis*, which was consistent with studies of *Pinus sylvestris L* and hyperaccumulator *S. nigrum* [23, 24]. Petiole was the transportation channel of water and nutrient from leaf to stem [25]. By speeding up the transportation of water and nutrients, the shorten petiole of *V. bonariensis* elevated the resistance to Cd stress. For leaf chlorosis, there existed two possible reasons: one was that the certain amount of Cd in the leaves rendered chlorophyll destruction and leaves chlorosis; the other was that due to the serious affliction to the root system and the malfunction of water transportation system, water shortage occurred in leaves. The above speculation was supported by the decrease of chlorophyll content, petiole length, leaf area, root length and number in *V. bonariensis* (Fig. 3c).

### Cd accumulation and transportation in *V. bonariensis*

Typically, most positively charged HM ions tended to bind negative-charged compounds in tissues. Consequently, these ions accumulated in roots [26]. In our results, Cd accumulation in roots was significantly higher than that in aboveground parts, for the retention on Cd^2+^ in root system. Through Cd enrichment in root, Cd^2+^ were prevented from interrupting photosynthesis and metabolism in plants. Consequently, botanical survival under stress could be possible. The biomass of *V. bonariensis* were significantly reduced in 100 mg/kg Cd solution. This very consistency was significantly higher than the critical concentration of *S. nigrum*, Cd stress over 25 mg/kg inhibited the growth of *S.nigrum* and decreased its biomass (Additional file 1: Figure S1) [27]. BCF indicated the transportation difficulty of HM elements in soil plant system [27]. The transportation and accumulation level of HMs from plant roots to the upper part of the plant were assessed by the BTF. For a hyperaccumulator, the BCF and BTF should be greater than 1 (Fig. 2a, b). The results proved that *V. bonariensis* showed no sign of hyperaccumulator. The absorption amount of Cd was 31.66 μg/pot in *V. bonariensis* (Fig. 2c). By contrast, Cd hyperaccumulator *Bidens pilosa L.* was only 17.92 μg/pot [27].

Based on the research results, *V. bonariensis* did not meet the standard of Cd hyperaccumulator. However, it had strong tolerance and absorption ability to Cd. A large amount of Cd was accumulated in roots of *V. bonariensis* under Cd stress. Consequently, the reduced amount of Cd in leaves and other sensitive organs cast lighter toxic effects on plants. This was consistent with the results of the study that *Lonicera Japanica Thunb* [28] and *Helianthus annuus* [29]. In brief, *with* rapid growth capability, large biomass, strong Cd tolerance and absorption ability, *V. bonariensis* possessed potential application value in the remediation of Cd pollution.

### Effects of cd stress on cell wall and cell membrane of *V. bonariensis*

The cell wall weighed significantly in botanical HM defense and detoxification [30]. As the first HMs barrier, it was firstly affected by Cd^2+^. The cell wall and carbohydrates protected Cd from entering roots by bounding it to the pectin site, which prevents HM ions from entering the protoplasm of the cell and protecting it from harm [31]. When exposed to HMs, the cell wall could activate hundreds of specific stress-responsive signaling proteins to protect the cell from crashing into the protoplast on susceptible sites. The lignin had a strong adsorption capacity for HM ions because it means a lot of radical groups, such as oxhydryl, methoxy and carbonyl group. The particle size of lignin was small, which was beneficial to the exposure of more radical groups and more HM ions could be adsorbed [32]. In our results, there were 7 GO entries with cell wall tissue correlation, which suggested that *V. bonariensis* might increase its tolerance to HMs by combining the root cell wall with Cd^2+^. The lignin relating to phenylpropanoid pathway could reinforce specialized cell walls [33]. All 18 DEGs associated with lignin synthesis was up-regulated. The content of lignin under Cd stress was significantly higher than that of the control. This indicated that the cell wall of *V. bonariensis* might be reinforced and substantial Cd^2+^ in soil be absorbed under Cd stress.

The cell membrane served as the second barrier against trespassing of HMs. Cd was an important mutagen of plasma membrane peroxidation. MDA was induced by more ROS produced under Cd stress, causing membrane lipid peroxidation as well as destroying membrane ion channel structure [34]. The membrane lipid peroxidation in the cell of *V. bonariensis* was demonstrated by the significant elevation of MDA in leaves and roots under Cd stress.

### Effects of cd stress on ROS scavenging system in *V. bonariensis*

Previous studies have shown that HM may injure plants by two biological pathways [35, 36]. On one side, the HM stress oxidation inhibited the activity of protective enzyme. The main biological macromolecules such as proteins and nucleic acids were destroyed by the induced free radicals. On the other side, when absorbed into the plant, HM ion not only combined with nucleic acids, proteins, enzymes and other substances, but also supplant some specific elements exercising the function of enzymes and proteins, which make the related enzymes and proteins denature or reduce their activities. Under Cd stress, the ROS scavenging system played a vital role in plants. As the primary defense enzyme purging ROS in cell, SOD converted O_2_^−^ disproportionation into H_2_O_2_ and eliminated -OH by catalyzing the Fenton reaction [37]. Cd stress is thought to elevate SOD activity in plants, but this promotion to SOD activity vary with HM treatment concentration and duration, plant species, and plant size [38]. In our study, the SOD activity in leaves and roots decreased under Cd treatment, it was speculated that excessive Cd^2+^ or stress time could inhibit the activity of SOD. Under Cd stress, the activities of POD, CAT and APX elevated in leaves of *V. bonariensis*. However, the results were opposite in roots. For its contact with soil, the roots were primarily susceptible to HM. Consequently, the stress level in roots was higher than that in leaves. When antioxidant enzyme activities in the root were hampered, the very activities in leaves continued coping with Cd stress. In the up-regulated GO enrichment categories relating to oxidative reactions, the enrichment degree of ‘oxidation-reduction process’, ‘oxidoreductase activity’ and ‘catalytic activity’ were high. The result showed that the oxidative reactions might be activated in response to Cd stress. By gearing the antioxidant system up, *V. bonariens* refrained from HM damage.

### Effects of cd stress on chelating reaction in *V. bonariensis*

Upon exposure to HMs, plants synthesized diverse metabolites, particularly specific amino acids, such as PRO and histidine, peptides (glutathione and phytochelatins (PC) etc), and organic acids [39]. These matters mentioned above interacted with Cd^2+^ to form chelates, such compounds reduced the concentration of Cd^2+^ in soil. Furthermore, direct contact between Cd^2+^ and organelles were eliminated. Thereby the toxicity of Cd was reduced in soil.

Amino acid, as one of the plant’s fundamental metabolites, counted great deal in the alleviation of HM stress. It served as integral part of the involved coenzyme and ligand in the metal complexation [40]. Cd stress resulted in a significant increase in the content of some amino acids, which might be a plant specific genetic trait. PRO regulated plant osmotic/redox reactions and participated in the metal complexation. In our study, Cd stress increased the accumulation of PRO in aboveground part by 29.76%, whereas in roots the percentage was 4.68%. Similarly, the amount of PRO in leaves was higher than that in roots of *Bacopamonnieri* under Cd stress [41].

For the great affinity to HMs, PCs chelate various HMs to deactivation [42]. When the Cd^2+^ entered the cytoplasm through the cell wall and cell membrane, it combined with PC to form LMW complex, which was transferred into vacuole under the action of htm1 membrane transportation protein. Then HMW complexes were synthesized by LMW and Cd, eventually immobilized in vacuole. The HMW complexes were less toxic to plants. PCs was a sulfhydryl polypeptide composed of cysteine, glutamic acid and glycine. As the precursor of PC synthesis, GSH composed some sulfur-containing compounds in root cells and Cd^2+^ to form stable chelates [43]. In our study, 76% of the DEGs involved in the ‘glutathione metabolism’ pathway was up-regulated. GSH content increased (Fig. 6). In this result, the promotion of PC content was predictable.

The organic acids of plants, such as oxalic acid, malic acid and citric acid, could be transformed the toxic Cd into low toxic or non-toxic form by chelating, promoting the tolerance of plants [44]. The pathways of organic acids in *V. bonariensis* were significant up-regulated in our results. It was estimated that the efficiency of organic acid synthesis was elevated. This promoted the binding of Cd^2+^ to organic acids in the cytoplasm or vacuoles, and alleviated the damage of HMs to *V. bonariensis*. The organic acids secretion capacity in Cd-tolerant plants such as *Rorippaglobosa* was far greater than that in non-tolerant plants *Rorippa* [45]. The improvement of organic acid raised the soil acidity of the rhizosphere as well as reducing the Cd uptake by plants. Exposed to low concentration of Cd, *Bechmerianivea* could secrete organic acids in its rhizosphere. With Cd chelating, the consistency of Cd^2+^ around the rhizosphere of *Bechmerianivea* decreased [46]. In transcriptome data of *V. bonariensis* under Cd stress, there were three pathways relating to organic acid metabolism among top-ten up-regulated pathway, including ‘Citrate cycle’, ‘Glyoxylate and dicarboxylate metabolism’ and ‘alpha-Linolenic acid metabolism’. The result proved the significant function of organic acid metabolism in *V. bonariensis* under Cd stress.

### Effects of cd stress on secondary metabolites of *V. bonariensis*

Although minor to plant growth and development, secondary metabolites were often involved in environmental stress [47]. The phenolic metabolism was an important process in plants’ secondary metabolism. Under Abiotic stresses, a large number of phenolic compounds was induced to form mechanical barriers in order to prevent osmotic stress, or to remove excessive amounts of ROS in cells [48]. Most of the phenolic compounds were composed of flavonoids, simple phenols and quinones. The flavonoids, as an important botanical antioxidant, played a key role in resistance to stress [49]. The synthesis efficiency of flavonoids could be improved by the activation of peroxidase under Cd stress [50]. CHS and ANS relating to flavonoid biosynthesis belonged to the family of oxidoreductases. CHS was the first enzyme to spur phenylpropane metabolic pathway to conduct flavonoids synthesis. It served as a natural defense enzyme as well as a synthetic intermediate in plants [51]. Anthocyanin was a strong antioxidant, it can alleviate the toxicity of oxygen free radicals in plant cells. In our results, only one gene down-regulated in 5 CHS and 9 ANS genes, respectively. The content of anthocyanidin, the activity of CHS and ANS were significantly elevated. The results showed that CHS and ANS genes may play an important regulated role in *V. bonariensis* resist the damage of Cd stress.

The phenylpropanoid biosynthesis has been demonstrated to contribute to various aspects of plant biotic and abiotic responses [52]. The improvements of phenolic compound content under abiotic stress, particularly with respect to phenylpropanoid, have been extensively described [53]. In *Lupinus luteus L*., the phenylpropanoid pathway metabolites elevated Pb tolerance in its roots [54]. Occupied the third place in up-regulated pathway, the ‘Phenylpropanoid biosynthesis’ was essential under Cd stress in *V. bonariensis* (Table 3).

### Effects of cd stress on transpiration and photosynthesis in *V. bonariensis*

Under Cd stress, the Tr of *V. bonariensis* decreased. The entrance of Cd^2+^ in guard cells through Ca^2+^ ion channel might induce stomatal closure through ‘Abscisic acid’ (ABA) pathway and inhibit transpiration in plants. These elements disturbed stomatal opening. In addition, Cd stress reduced the length and number of roots, limiting water intake (Fig. 3a). Therefore, the leaf area of *V. bonariensis* decreased to maintain water in cell. Similarly, Tr and leaf area of *Brassica juncea* were hampered under Cd stress [55].

Cd^2+^ damaged nucleoli in the cell of root tip, precluding the synthesis of RNA and the activities of RNAase, ribonuclease and proton pump. This process decreased nitrate reductase activity, reduced the uptake and transportation of nitrate from the root to the aboveground part. With the HM ions shifted to the upper part of plants, dwarfism and decreased biomass occurred. The upward transportation of nutrients was forestalled by the factors mentioned above. The lack of nutrients hindered photosynthesis and the growth of the plants. This action decreased photosynthetic rate, destroyed photosynthetic organs, damaged photosynthetic systems, disturbed carbon dioxide fixation, and even death [56].

In our experiment, Pn and Gs decreased whereas CO_2_ concentration (Ci) increased (Fig. 7). Stomatal and non-stomatal components were closely related to the Pn decrease [57]. Besides, as a non-stomatal limitation, chlorophyll decomposition accounted for the decline of Pn. The results illustrated that under Cd stress photosynthesis in *V. bonariensis* leaves were abated. As a result of Gs decline, CO_2_ supply decreased. The non-stomatal factors that hindered the utilization of CO_2_, resulted in the accumulation of intercellular CO_2_. Non-stomatal factors took a great to injure the chloroplast of *V. bonariensis* under stress and decrease the photosynthetic cell activity.

In ‘Photosynthesis-antenna proteins’ pathway, only one gene encoding LHC was up-regulated. As a peripheral antenna system, antenna proteins in LHC elevated the efficiency of absorption of light energy [58]. Most of the DEGs associated with the ‘Photosynthesis’ were down regulated, indicating that Cd stress arose disorders in photosynthetic responses. Cd stress prevented light harvesting, electron transportation and carbon assimilation efficiency during photosynthesis in *V. bonariensis*. This was consistent with previous studies on the response of Maize to Pb [59]. These physiological and molecular changes suggested that down-regulation of the photosynthetic pathway might be a responsive step in *V. bonariensis* under Cd stress.

## Conclusions

In this study, the Cd tolerance of *V. bonariensis* was exhaustively analyzed on physiological and molecular scales. The large-scale transcriptional data set of *V. bonariensis* in response to Cd stress was firstly obtained, *V. bonariensis* was identified as a HM tolerant plant in the first time. ROS system, transpiration and photosynthetic, secondary metabolism and Chelating reaction in *V. bonariensis* under Cd stress were understood by transcriptional data. Some promising DEGs that aided the tolerance to Cd in plants were found. In conclusion, our research will be beneficial for understanding the mechanism of Cd resistance in *V. bonariensis*. The clues for further studies on the relationships between plants and HMs in other *Verbena* plants were listed.

## Methods

### Materials and germination experiment design

*V. bonariensis* seeds were purchased from Germany Benary seed company. The treatment solution was prepared with deionized water and CdCl_2_•2.5H_2_O. The concentrations were 5 mg/L, 10 mg/L, 20 mg/L, 50 mg/L, 100 mg/L, 150 mg/L, 200 mg/L. The Reverses Osmosis (RO) pure water was used as controlling group. The two sheets of filter paper were placed in a culture dish of 9 cm in diameter. Ten milliliters of treatment solution were added to saturate filter paper. The wet filter paper was used as a bed for germination. Thirty seeds were placed in each dish, sorted to a total of three replicates. All experiments were performed three times to ensure biological repetitions. The dishes were placed in the incubator (16 h photoperiod, 25 °C/16 °C day/night temperature). The culture dish was sealed with a sealing film to keep humidity. The germination condition was observed daily until the germination of the controlling group revealed unchanged. The incubation time was about 2 weeks.

Germination rate (%) = number of germinated seeds within 7 days/ total seed * 100%;

GI = ∑Gt /Dt (Gt for germination number of t days, Dt for corresponding days);

VI = GI * biomass (the biomass was the fresh weight of individual seedling) [60].

### Cd treatment

The robust plants with health growth were selected for soil Cd stress treatment. Seedling age was 30-day old. One seedling was planted in each plastic flowerpot. Fifteen pots were involved in each treatment. The perlite and peat soil (PINDSTRUP, DK) were mixed evenly at 1:1 (v: v) and sterilized with right amount of carbendazim. With 15-day air-dry, the mixed soil was put into the circular plastic flowerpot (d = 12 cm) on the standard of 1 kg per pot. The soil moisture content was controlled as 70% using the RO water (about 180 ml).

(1) Cd treatment of different concentration was designed. In order to obtain detailed and accurate data, the content of Cd in *V. bonariensis* was measured. A total of 50 mg/kg (T1), 100 mg/kg (T2), 200 mg/kg (T3), 300 mg/kg (T4), and 400 mg/kg (T5) were set up for 5 Cd concentrations. The CK were treated without Cd solution. Three seedlings were planted in each pot, each treatment with 10 pots. During the experiment, plants and soil samples were collected every 10 days for 4 times.

(2) Cd treatment for RNA-Seq was arranged. The experiment was repeated three times with a total of 90 pots. The CK were treated without Cd solution. The concentration of Cd in the experimental group was 100 mg/kg. CdCl_2_·2_1_/2H_2_O and RO water were mixed to form 150 ml Cd solution with different concentrations. The mix solution was applied evenly into the corresponding flowerpot on the first day. After 20 days under Cd stress, the roots were harvested. The root samples were immediately frozen in liquid nitrogen and stored at − 80 °C. In this experiment, the seedings were grown in ambient conditions with a photoperiod of 14 h at 25 °C and a relative humidity of 75%.

#### Determination of cd in plants and soils

Plant (root, stem and leaf) and soil samples were collected and dried. These samples were crushed. Then the samples were digested by HNO_3_-HCLO_4_ per 0.5 g. The atomic absorption flame spectrophotometer was applied to determine the content of Cd. All of the above experiments were repeated three times.

BCF = C_plant_ /C_soil_;

BTF = C_overground part_ /C_subterranean part_;

In the formula, C_plant_ was the concentration of HM in a part (root, stem and leaf) of the plant (mg/kg); C_soil_ was the concentration of corresponding HM element in soil (mg/kg); C_overground part_ was the concentration of HM in the upper part of the plant (mg/kg); C_subterranean part_ was the concentration of HM in lower parts of plant (mg/kg) [61].

#### Determination of morphological characteristics and physiological indexes

The leaf and root samples were collected. Some of them was used for morphological measurement. Others were immediately frozen in liquid nitrogen and stored at − 80 °C for physiological measurements. Morphological features were measured according to Bai et al. [62]. Histochemical staining of ROS (H_2_O_2_ and O_2_^−^) methods referred to Wang et al. [63]. The content of MDA was determined by thiobarbituric acid colorimetric assay proposed by Cakmak and Marschner [64]. Nitro-blue tetrazolium photoreduction method, Guaiacol method and Ultraviolet absorption method were used to determine the activity of SOD, POD and CAT, respectively [65]. APX activity was determined by reference to Nakano et al. and the OD_290_ changes were measured per minute [66]. The activity of GSH and the content of PRO were determined according to Quessada et al. [67] and Bates et al. [68], respectively. Blade gas exchange parameter was measured at nine o’clock in the morning and the endogenous light intensity was 800 μmol quanta m^− 2^ s^− 1^ [69].

The content of lignin and anthocyanidin in roots was measured by referring to the method of Chang XF et al. [70] and Dedaldechamp et al. [71], respectively. The activity of chalcone synthase and anthocyanidin synthase in roots were measured by A Special Kit for the determination of Plant anthocyanin activity in TSZ Company of the United States.

### RNA isolation, library construction and RNA-Seq analysis

Roots with two different treatments were collected from *V. bonariensis*. RNA-Seq analysis were performed with three replicates. Total RNA isolation was performed with Trizol Reagent (Invitrogen) according to the manufacturer’s protocol. RNA purity and concentration were checked using the NanoPhotometer® spectrophotometer (IMPLEN, CA, USA) and Qubit® RNA Assay Kit in Qubit® 2.0 Flurometer (Life Technologies, CA, USA), respectively. In brief, mRNA was enriched from total RNA using poly-T-oligo-attached magnetic beads. as template to synthesize double stranded cDNA, mRNAs were purified with AMPure XP beads (Beckman Coulter, Beverly, USA). the purified double stranded cDNA was subjected to terminal repair and then supplemented with A tail to connect sequencing connector. In order to select cDNA fragments of preferentially 150~200 bp in length, the library fragments were purified with AMPure XP system. At last, PCR was performed with Phusion High-Fidelity DNA polymerase, universal PCR primers and Index (X) Primer. The qualifed libraries assessed on the Agilent Bioanalyzer 2100 system were sequenced on an Illumina Hiseq2500 platform.

### Raw sequence procession, assembly and functional annotation

After filtering, clean data was obtained. Transcriptome de novo assembly was accomplished using Trinity with min-kmer-cov set to 2 by default. A BLASTx search was used for further functional annotation of the unigenes against the NCBI non-redundant protein sequences (Nr), NCBI nucleotide sequences (Nt) and Swiss-prot with an *E*-value of ≤10^− 5^, while compared with euKaryotic Ortholog Groups (KOG) with an *E*-value of ≤10^− 3^. The HMMER3 program was used to assign Protein family (Pfam) with an *E*-value = 0.01. GO using Blast2GO v2.5 program with an *E*-value = 1e^− 6^. According to the KEGG database, pathway assignments were carried out using BLASTx with *E*-value = 1e^− 10^.

### The analysis of differential expression

The input data of gene differential expression is the read count data obtained in the analysis of gene expression level. The read count data were standardized by trimmed mean of *M*-value. DESeq [72] was used to carry out the differential analysis. The Benjamini and Hochberg’s approach [73] to control the False Discovery Rate (FDR) was applied to adjust *p*-value of the results. DEGs screening condition was FDR < 0.05.

### qRT-PCR validation

To further validate the DEGs identified in analysis of the RNA-Seq data, ten DEGs were selected randomly to perform qRT-PCR analysis with three replicates. The RNA from the isolated RNA sequencing samples mentioned above. The qRT-PCR reaction system was showed in Table 4. The PCR cycling conditions was 95 °C for 30 s, followed by 40 cycles of 95 °C for 15 s and 60 °C for 30 s. The gene-specific primers were designed by Primer 5. The *Actin* gene (F: GAAAGATGGCTGGAAGAGGG; R: GCTATGAACTCCCTGATGGTC) was served as a reference control to detect expression level of 10 DEGs. The primer sequences were shown in (Additional file 8: Table S3). The data was analyzed using the 2^-ΔΔCT^ method.

**Table 4:** qRT-PCR reaction system

Reagent	Quantity
SsoFast EvaGreen supermix	10 μl
cDNA template	2 μl
Forward Primer (10uM)	0.8 μl
Reverse Primer (10uM)	0.8 μl
RNase/Dnase free water	6.4 μl
Total	20 μl

### Statistical analysis

The experimental data were statistically analyzed by SPSS17.0 (SPSS Inc., Chicago, USA). The significance test of difference was made by the LSD method, significance level setting *P* = 0.05.

## Supplementary information

**Additional file 1: Figure S1.** Changes of *Verbena bonariensis* biomass under Cd different concentration stress.

**Additional file 2: Figure S2.** Changes of *Verbena bonariensis* morphological indexes under Cd different concentration stress. (a) Leaf area; (b) petiole long; (c) Plant height; (d) Root length; (e) Number of lateral roots.

**Additional file 3: Figure S3.** Changes of *Verbena bonariensis* physiological indexes under Cd different concentration stress. (a) POD activity; (b) SOD activity; (c) APX activity; (d) Soluble sugar content; (e) Soluble protein content; (f) PRO content; (g) GSH activity; (h) MDA activity.

**Additional file 4: Figure S4.** Unigenes notes success statistics in each database.

**Additional file 5: Figure S5.** Unigenes classified statistics based on GO annotations.

**Additional file 6: Table S1.** DEGs encoding lignin synthesis in ‘phenylpropanoid biosynthesis’ pathway.

**Additional file 7: Table S2.** DEGs encoding chalcone synthase (CHS) and anthocyanidin synthase (ANS).

**Additional file 8: Table S3.** The primers of 10 DEGs and different parameters derived from qRT-PCR analysis.

## Data Availability

The raw sequencing data have been submitted to the NCBI Sequence Read Archive database with accession number GSE113569.

## Abbreviations

ABA: Abscisic acid
ANS: Anthocyanidin synthase
APX: Ascorbate peroxidase
BCF: Bioaccumulation factor
BP: Biological processes
BTF: Translocation factor
CAT: Catalase
CC: Cell Component
Cd: Cadmium
Chla: Chlorophyll a
Chlb: Chlorophyll b
CHS: Chalcone synthase
Ci: CO_2_ concentration
CK: control group
DEGs: Differentially expressed genes
FDR: False discovery rate
GI: Germination index
GO: Gene ontology
Gs: stomatal conductance
GSH: Glutathione
HM: heavy metal
KEGG: Kyoto Encyclopedia of Genes and Genomes
KOG: EuKaryotic orthologous groups
MDA: Malondialdehyde
MF: Molecular function
MT: Metallothionein
Nr: NCBI non-redundant protein sequences
Nt: NCBI nucleotide sequences
PC: Phytochelatin
Pfam: Protein family
Pn: Net photosynthetic rate
POD: Peroxidase
PRO: proline
qRT-PCR: Quantitative real-time polymerase chain reaction
RNA-Seq: RNA-Sequence
RO: Reverses Osmosis
ROS: Reactive oxygen species
SOD: Superoxide dismutase
Tr: transpiration rate
VI: Vigor index
