# Supplementary material for: Transcriptomic analysis of Verbena bonariensis roots in response to cadmium stress
Source: BMC Genomics. 2019 Nov 20;20:877. doi: 10.1186/s12864-019-6152-9 (PMC6868873; doi:10.1186/s12864-019-6152-9)
Supplement: Supplementary file 1 — Additional file 1: Figure S1. Changes of Verbena bonariensis biomass under Cd different concentration stress. [file 12864_2019_6152_MOESM1_ESM.docx]

**Additional file 1:**


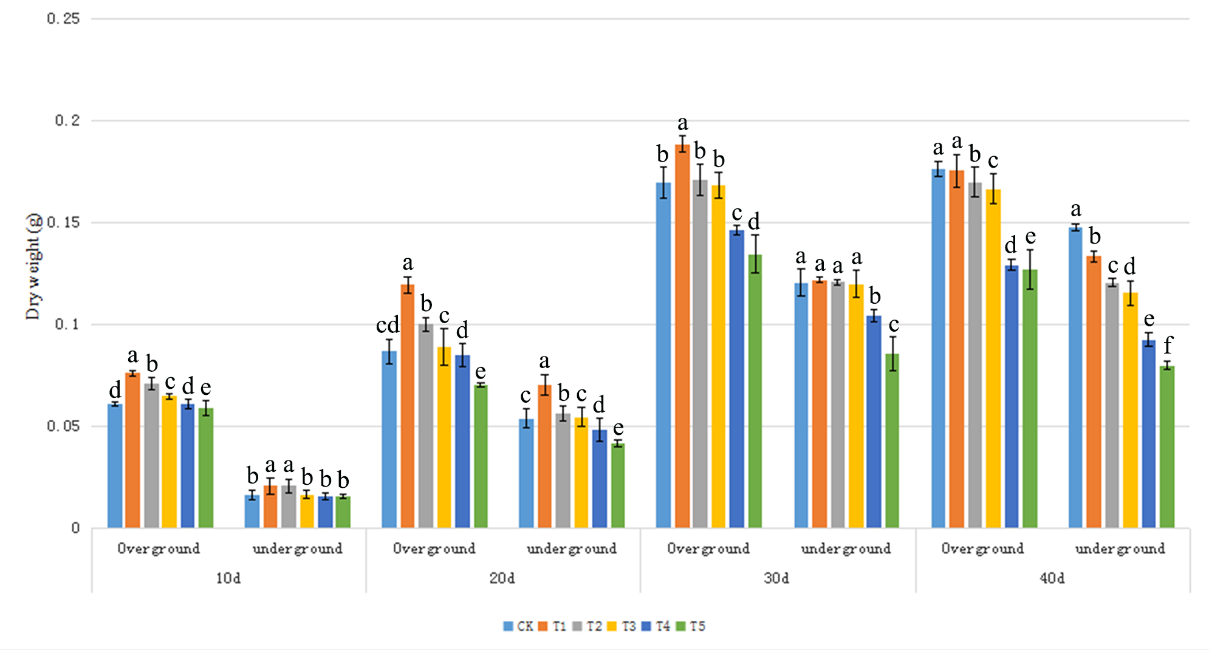


**Figure S****1** Changes of *Verbena bonariensis* biomass under Cd different concentration stress.
