# Supplementary material for: Transcriptomic analysis of Verbena bonariensis roots in response to cadmium stress
Source: BMC Genomics. 2019 Nov 20;20:877. doi: 10.1186/s12864-019-6152-9 (PMC6868873; doi:10.1186/s12864-019-6152-9)
Supplement: Supplementary file 2 — Additional file 2: Figure S2. Changes of Verbena bonariensis morphological indexes under Cd different concentration stress. (a) Leaf area; (b) petiole long; (c) Plant height; (d) Root length; (e) Number of lateral roots. [file 12864_2019_6152_MOESM2_ESM.docx]

**Additional file 2:**


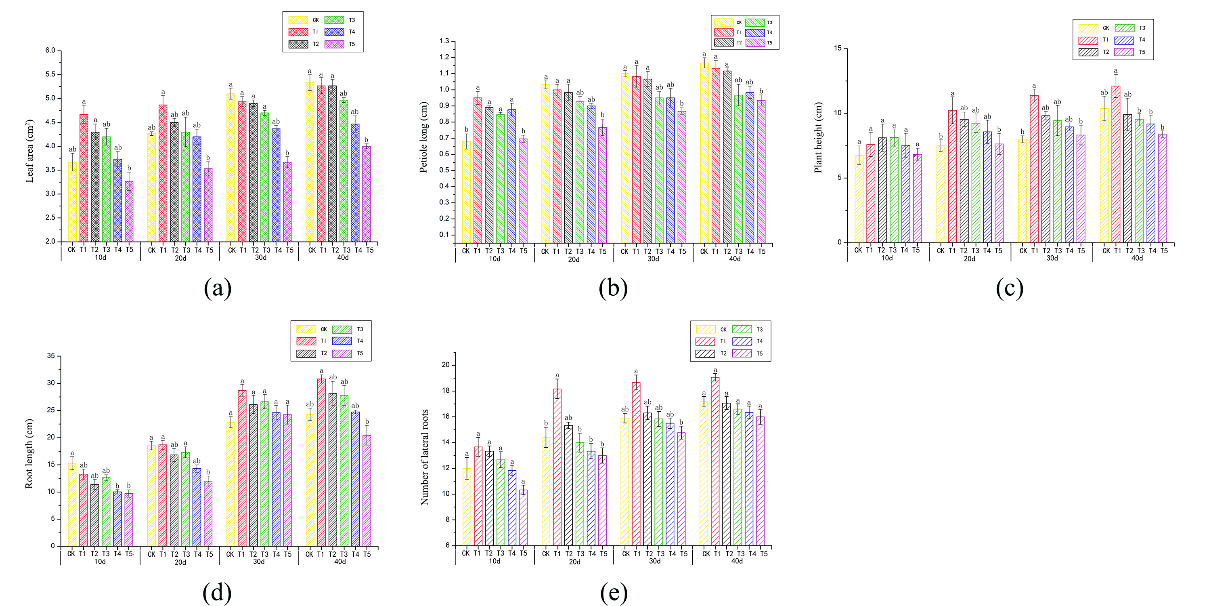


**Figure S2** Changes of *Verbena bonariensis* morphological indexes under Cd different concentration stress. (a) Leaf area; (b) petiole long; (c) Plant height; (d) Root length; (e) Number of lateral roots.
