# Supplementary material for: Transcriptomic analysis of Verbena bonariensis roots in response to cadmium stress
Source: BMC Genomics. 2019 Nov 20;20:877. doi: 10.1186/s12864-019-6152-9 (PMC6868873; doi:10.1186/s12864-019-6152-9)
Supplement: Supplementary file 3 — Additional file 3: Figure S3. Changes of Verbena bonariensis physiological indexes under Cd different concentration stress. (a) POD activity; (b) SOD activity; (c) APX activity; (d) Soluble sugar content; (e) Soluble protein content; (f) PRO content; (g) GSH activity; (h) MDA activity. [file 12864_2019_6152_MOESM3_ESM.docx]

**Additional file 3:**


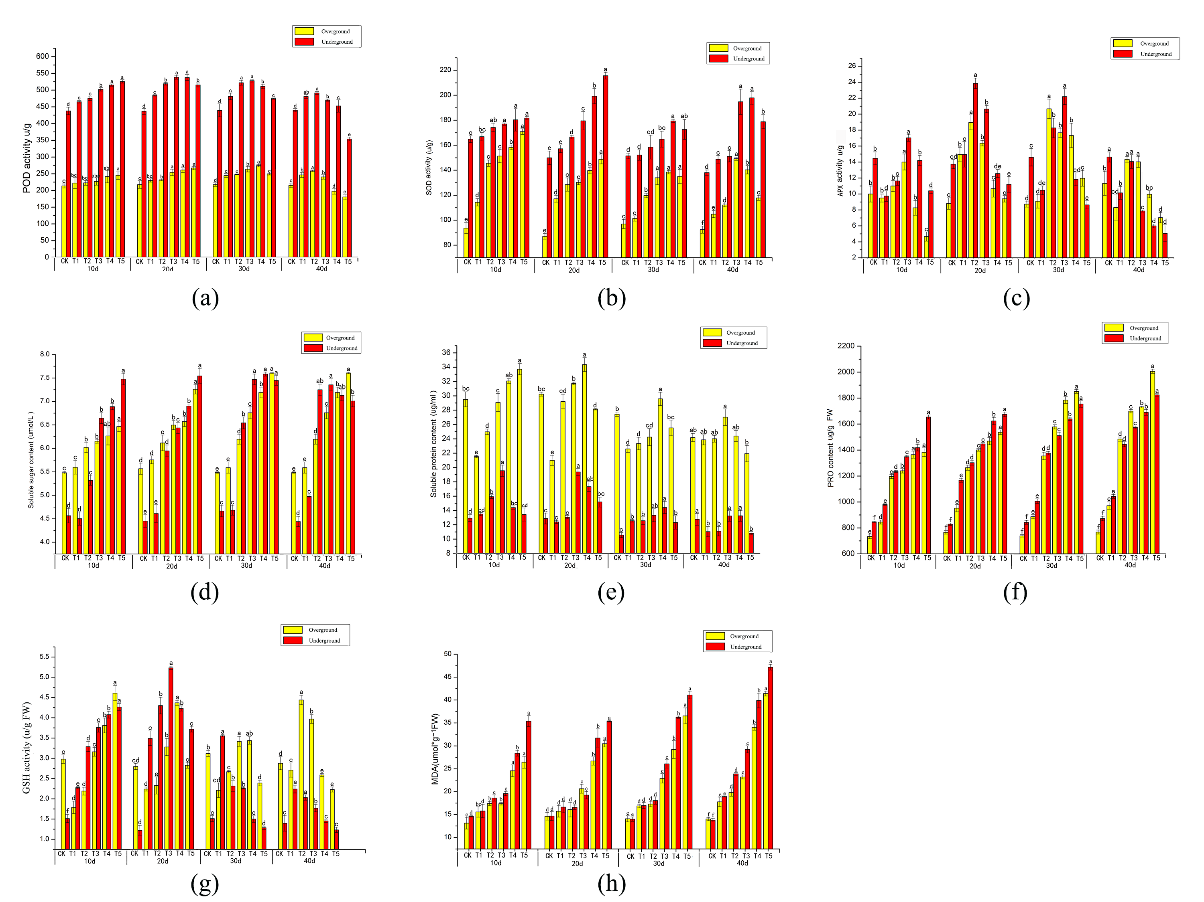


**Figure S3** Changes of *Verbena bonariensis* physiological indexes under Cd different concentration stress. (a) POD activity; (b) SOD activity; (c) APX activity；(d) Soluble sugar content; (e) Soluble protein content; (f) PRO content; (g) GSH activity; (h) MDA activity.
