# Supplementary figures and images for: Transcriptomic analysis of Verbena bonariensis roots in response to cadmium stress
Source: BMC Genomics. 2019 Nov 20;20:877. doi: 10.1186/s12864-019-6152-9 (PMC6868873; doi:10.1186/s12864-019-6152-9)

**Additional file 4:**


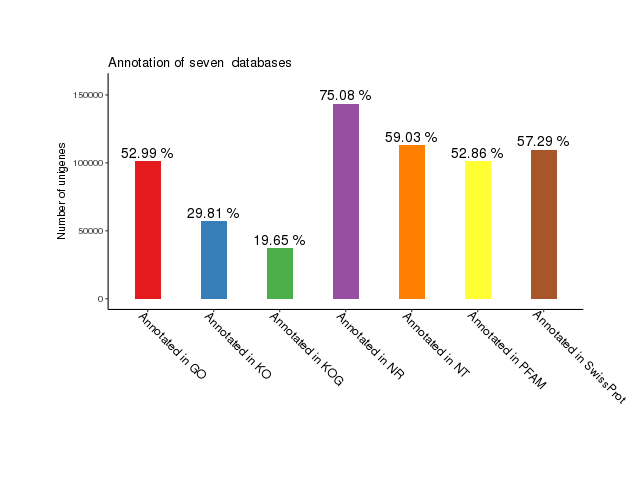


**Figure S4** Unigenes notes success statistics in each database.

Supplement: Supplementary file 4 — Additional file 4: Figure S4. Unigenes notes success statistics in each database. [file 12864_2019_6152_MOESM4_ESM.docx]

**Additional file 5:**


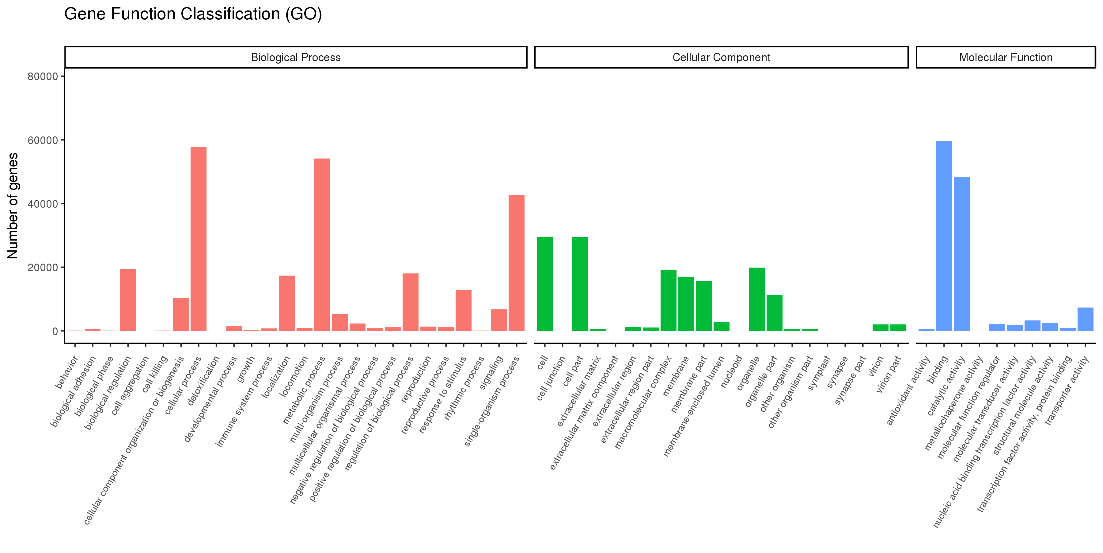


**Figure S5** Unigenes classified statistics based on GO annotations.

Supplement: Supplementary file 5 — Additional file 5: Figure S5. Unigenes classified statistics based on GO annotations. [file 12864_2019_6152_MOESM5_ESM.docx]
