# Supplementary material for: Transcriptomic analysis of Verbena bonariensis roots in response to cadmium stress
Source: BMC Genomics. 2019 Nov 20;20:877. doi: 10.1186/s12864-019-6152-9 (PMC6868873; doi:10.1186/s12864-019-6152-9)
Supplement: Supplementary file 6 — Additional file 6: Table S1. DEGs encoding lignin synthesis in ‘phenylpropanoid biosynthesis’ pathway. [file 12864_2019_6152_MOESM6_ESM.docx]

**Additional file 6:**

**Table S1** DEGs encoding lignin synthesis in ‘phenylpropanoid biosynthesis’ pathway

| Gene ID | NR Description | UP_DOWN | log2ratio | FDR |
| --- | --- | --- | --- | --- |
| Cluster-9033.38247 | lignin-forming anionic peroxidase-like | UP | 1.7127 | 0.01997 |
| Cluster-9033.25428 | lignin-forming anionic peroxidase-like | UP | 3.866 | 0.009994 |
| Cluster-9033.158045 | lignin-forming anionic peroxidase-like | UP | 5.0449 | 4.67E-18 |
| Cluster-9033.158044 | lignin-forming anionic peroxidase-like | UP | 6.5997 | 1.81E-05 |
| Cluster-9033.38253 | lignin-forming anionic peroxidase-like | UP | 2.9743 | 7.02E-09 |
| Cluster-9033.57057 | lignin-forming anionic peroxidase-like | UP | 2.9827 | 2.26E-62 |
| Cluster-9033.57056 | lignin-forming anionic peroxidase-like | UP | 3.6594 | 5.71E-14 |
| Cluster-9033.38258 | lignin-forming anionic peroxidase-like | UP | 2.1568 | 0.001626 |
| Cluster-9033.38254 | lignin-forming anionic peroxidase-like | UP | 0.83904 | 0.03929 |
| Cluster-9033.38261 | lignin-forming anionic peroxidase-like | UP | 2.349 | 4.49E-11 |
| Cluster-9033.38262 | lignin-forming anionic peroxidase-like | UP | 2.3387 | 2.57E-06 |
| Cluster-9033.69850 | lignin-forming anionic peroxidase-like | UP | 3.9277 | 1.32E-38 |
| Cluster-9033.38249 | lignin-forming anionic peroxidase-like | UP | 1.6687 | 7.08E-06 |
| Cluster-9033.38248 | lignin-forming anionic peroxidase-like | UP | 3.3031 | 1.43E-07 |
| Cluster-9033.115967 | Lignin-forming anionic peroxidase | UP | 3.6323 | 2.24E-11 |
| Cluster-9033.38260 | lignin-forming anionic peroxidase-like | UP | 2.5654 | 7.12E-05 |
| Cluster-9033.57059 | lignin-forming anionic peroxidase-like | UP | 2.4727 | 1.17E-06 |
| Cluster-9033.57058 | lignin-forming anionic peroxidase-like | UP | 4.3592 | 6.75E-29 |
