# Supplementary material for: Transcriptomic analysis of Verbena bonariensis roots in response to cadmium stress
Source: BMC Genomics. 2019 Nov 20;20:877. doi: 10.1186/s12864-019-6152-9 (PMC6868873; doi:10.1186/s12864-019-6152-9)
Supplement: Supplementary file 7 — Additional file 7: Table S2. DEGs encoding chalcone synthase (CHS) and anthocyanidin synthase (ANS). [file 12864_2019_6152_MOESM7_ESM.docx]

**Additional file 7:**

**Table S2** DEGs encoding chalcone synthase (CHS) and anthocyanidin synthase (ANS)

|  | gene_id | NR Description | Regulation | log2ratio | FDR |
| --- | --- | --- | --- | --- | --- |
| CHS | Cluster-9033.155943 | chalcone synthase | UP | 5.33 | 2.79E-77 |
|  | Cluster-9033.9502 | chalcone synthase | UP | Inf | 0.001238 |
|  | Cluster-9033.8669 | chalcone synthase | UP | 3.48 | 2.24E-06 |
|  | Cluster-9033.132286 | chalcone synthase | UP | 1.22 | 1.36E-10 |
|  | Cluster-9033.36145 | chalcone synthase | DOWN | -2.7 | 0.017966 |
| ANS | Cluster-9033.47812 | anthocyanidin 3-O-glucosyltransferase 2-like | UP | 1.47 | 1.02E-05 |
|  | Cluster-9033.62224 | anthocyanidin 3-O-glucoside 2'''-O-xylosyltransferase-like isoform X1 | UP | 1.1 | 2.00E-08 |
|  | Cluster-9033.117260 | anthocyanidin 3-O-glucoside 6''-O-acyltransferase-like | UP | 3.88 | 0.002062 |
|  | Cluster-9033.100327 | anthocyanidin 3-O-glucoside 2'''-O-xylosyltransferase-like isoform X1 | UP | 1.53 | 1.46E-13 |
|  | Cluster-9033.48950 | anthocyanidin 3-O-glucosyltransferase 2-like | UP | 2 | 0.049556 |
|  | Cluster-9033.10279 | anthocyanidin 3-O-glucosyltransferase 5-like | UP | 4.36 | 6.05E-98 |
|  | Cluster-9033.157845 | leucoanthocyanidin dioxygenase-like | UP | 2.5 | 0.004414 |
|  | Cluster-9033.154043 | leucoanthocyanidin dioxygenase-like | UP | 3.26 | 1.62E-16 |
|  | Cluster-9033.89282 | anthocyanidin 3-O-glucoside 6''-O-acyltransferase-like | DOWN | -0.6 | 0.000593 |
