# Supplementary material for: Transcriptomic analysis of Verbena bonariensis roots in response to cadmium stress
Source: BMC Genomics. 2019 Nov 20;20:877. doi: 10.1186/s12864-019-6152-9 (PMC6868873; doi:10.1186/s12864-019-6152-9)
Supplement: Supplementary file 8 — Additional file 8: Table S3. The primers of 10 DEGs and different parameters derived from qRT-PCR analysis. [file 12864_2019_6152_MOESM8_ESM.docx]

**Additional file 8:**

**Table S3** The primers of 10 DEGs and different parameters derived from qRT-PCR analysis.

| Gene ID | KEGG Pathway ID | Primers (5’-3’) | Tm (°C) | Aplicon Length (bp) | Primer efficiency (%) | R^2^ |
| --- | --- | --- | --- | --- | --- | --- |
| Cluster-9033.52487 | Ko00480 | Forward: ACATACCTGTAATGCCCAAGG  Reverse: ATATCAGCTTCAGAATCAAAATCCAG | 57 | 1022 | 95.8 | 0.992 |
| Cluster-9033.67800 | Ko00480 | Forward: GAGCTAAACACAACCAAGACAAC  Reverse: TCGACGCCCTTTAGTTTGAG | 56 | 489 | 95.7 | 0.992 |
| Cluster-9033.72348 | Ko00480 | Forward: ACATACCTGTAATGCCCAAGG  Reverse: ATATCAGCTTCAGAATCAAAATCCAG | 57 | 1712 | 96.9 | 0.990 |
| Cluster-9033.54229 | Ko00020 | Forward: ACCTGAAGTTTCCAAGCCTAG  Reverse: ACCAGTTCCTTTGATGTCCG | 58 | 1463 | 98.1 | 0.994 |
| Cluster-9033.85519 | Ko00020 | Forward:  CACCATTTTGAGCTTTCCAGG  Reverse: AATGTTCCTCGTCTTGTCCC | 58 | 998 | 99.2 | 0.993 |
| Cluster-9033.80556 | Ko00196 | Forward: GAGAAGTCCCAGAAAACCTAGAG  Reverse: CTTGACCCAGTTGCCCAG | 57 | 340 | 101.5 | 0.991 |
| Cluster-9033.83697 | Ko00196 | Forward: GAGAAGTCCCAGAAAACCTAGAG  Reverse: CTTGACCCAGTTGCCCAG | 57 | 946 | 98.3 | 0.993 |
| Cluster-9033.81543 | Ko00196 | Forward: TTGAAGGTGAATGGAGTGGAC  Reverse: CAATCAACCTCGCAGAAATGG | 58 | 344 | 99.9 | 0.992 |
| Cluster-4192.0 | Ko00564 | Forward: AATCCTCAGCACTTTCGTCC  Reverse: TGCATTAGCGACCATAACGG | 56 | 1655 | 101.9 | 0.995 |
| Cluster-9033.124060 | Ko00906 | Forward: GTTTGATAAAGGTTGGTGCTGAG  Reverse: GGGCTCCAATTGCGAAATG | 57 | 1020 | 96.0 | 0.993 |
